# Acute Versus Chronic Eosinophilic Pneumonia: A Case Report

**DOI:** 10.7759/cureus.46257

**Published:** 2023-09-30

**Authors:** Maureen N Ikedinobi, Ezioma Gbujie

**Affiliations:** 1 Medicine, University of North Dakota School of Medicine and Health Sciences, Grand Forks, USA; 2 Internal Medicine, Sanford Health, Fargo, USA

**Keywords:** bal fluid, lung disease, chronic, acute, eosinophilic pneumonia

## Abstract

Eosinophilic pneumonia is a very rare form of interstitial lung disease. It is subdivided into acute and chronic types. Both types share some characteristics differences and similarities. We report two unique cases of acute eosinophilic pneumonia (AEP) and chronic eosinophilic pneumonia seen in 37- and 30-year-old males, respectively. Both cases occurred in the summer of the same year. There was no known association between the two patients except that they were both young males. We were able to compare the unique features of AEP and chronic eosinophilic pneumonia (CEP) and how these relate to the cases presented.

## Introduction

Eosinophilic pneumonia is a rare lung disease that is characterized by the accumulation of eosinophils in the lung’s parenchyma, blood peripheral, and bronchoalveolar lavage (BAL). Blood eosinophilia is defined as an eosinophilic count >500 ×10^9^ cells/L [1]. Bronchoalveolar lavage (BAL) fluid eosinophilia is defined by >5% of eosinophils in differential cell count [1]. Lung parenchyma eosinophilic infiltration is usually demonstrated on lung biopsy specimen [1].

Eosinophilic pneumonia, which is referred to as a primary eosinophilic disorder, is further divided into acute and chronic types [2]. These types differ moderately in terms of etiology, clinical features related to disease onset, treatment plan, and disease course [3]. We report a case of acute eosinophilic pneumonia in a 37-year-old male that occurred in the early summer months and a case of chronic eosinophilia in a 30-year-old male who presented in the late summer months. Both patients experienced different course of the disease and treatment plan.

## Case presentation

Case 1

On a summer evening, a 37-year-old male presented to the emergency department with rapid-onset hypoxia, and oxygen saturation on room air was 80%. The patient appeared lethargic but was responding to questions appropriately. On arrival, he was placed on 15 L of oxygen via a non-rebreather mask that raised oxygen saturation to 91%. Other presenting problems include rapid-onset chest pain, trouble breathing, and diaphoresis. Earlier in the day, the patient reported a near syncopal event, central chest pressure, and shortness of breath with a cough. Per the wife’s report, the patient had experienced rapid weakness, fatigue, chills, diaphoresis, and fever over the past two days. At the time of presentation, the patient was not vaccinated with COVID. He was a former smoker who quit smoking a couple of years ago but continues to chew tobacco. The patient had no significant medical history. On physical exam, a toxic-appearing patient was appreciated. On auscultation, normal heart sounds with tachycardia were noted. Bilateral decreased breath sounds, wheezing, and rales were present. Skin appeared pale. There was an absence of facial droop and slurred speech. His pulse rate was 108 bpm, and his blood pressure was 121/80 mmHg. Electrocardiogram (EKG) showed slight J-point elevation, normal QTc duration, and absence of ST depression. Laboratory workup showed mild leukocytosis and mild C-reactive protein elevation. The absolute number of eosinophils on the day of presentation was 500 cells/ml, which trended up to 800 cells/ml on the third day of admission (see Table 1).

**Table 1:** Hematologic findings seen on day one versus day three of a patient hospitalized from acute eosinophilic pneumonia (AEP) ESR: erythrocyte sedimentation rate, FEU: fibrinogen equivalent unit.

Hematologic findings	Hospital day 1	Hospital day 3	Reference range
WBC	13.4 K/µl	12.8 K/µl	4-11 K/µl
Eosinophil	0.5 K/µl; 500 cells/ml	0.8 K/µl; 800 cells/ml	0.7 K/µl
C-reactive protein	35.1 mg/L		0-8.0 mg/L
ESR	-	-	0-14 mm/hr
Procalcitonin	0.07 ng/mL	0.06 ng/ml	Less than 0.007 ng/ml
Lactic acid	1.4 mmol/L		0.5-2.2 mmol/L
Troponin I	0.004 ng/mL		0.033 ng/mL
D-dimer		0.65 µg/ml FEU	Less than 0.49 µg/ml FEU
Bronchoalveolar lavage eosinophil		24%	

**Figure 1:**
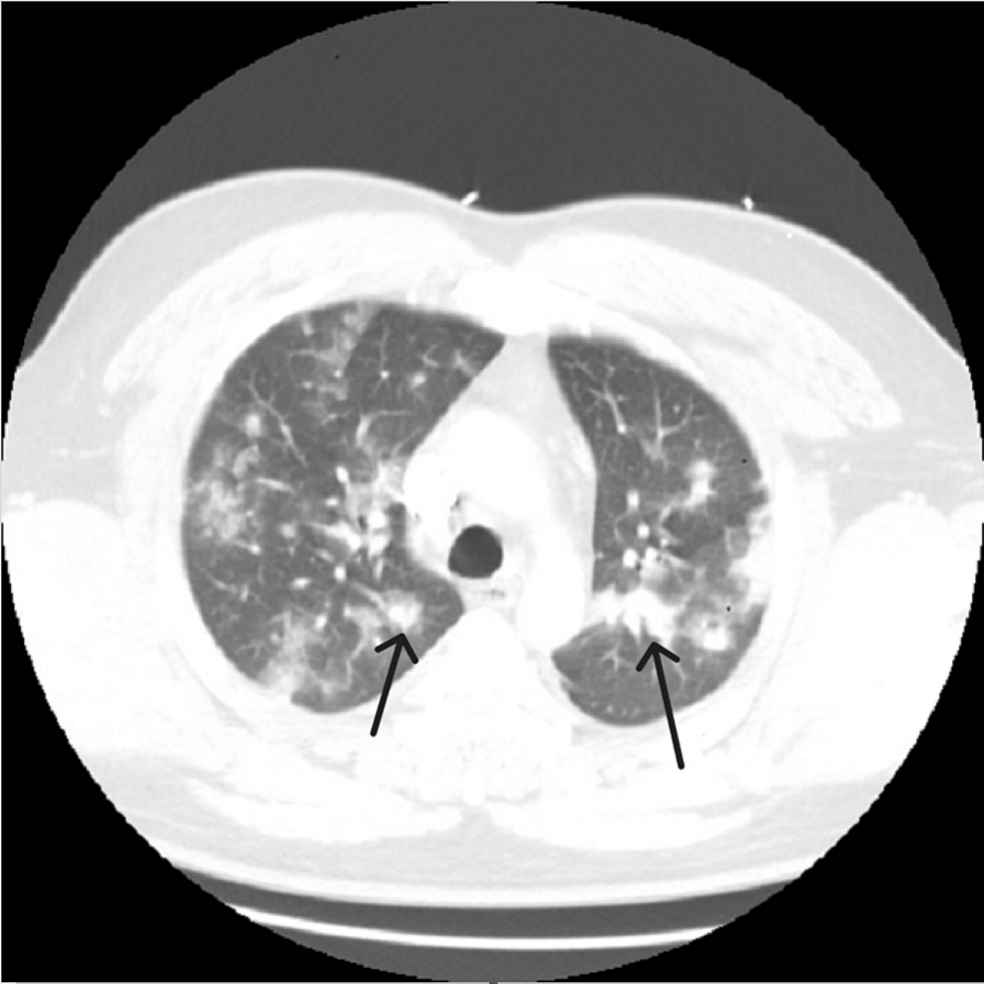
Chest computed tomography (CT) with ground-glass opacity, almost cannonball appearing (black arrows) in a case of acute eosinophilic pneumonia (AEP)

CT scan showed numerous nodular densities scattered throughout both lungs with ground-glass opacities, almost cannonball appearing (see Figure 1). 

**Figure 2:**
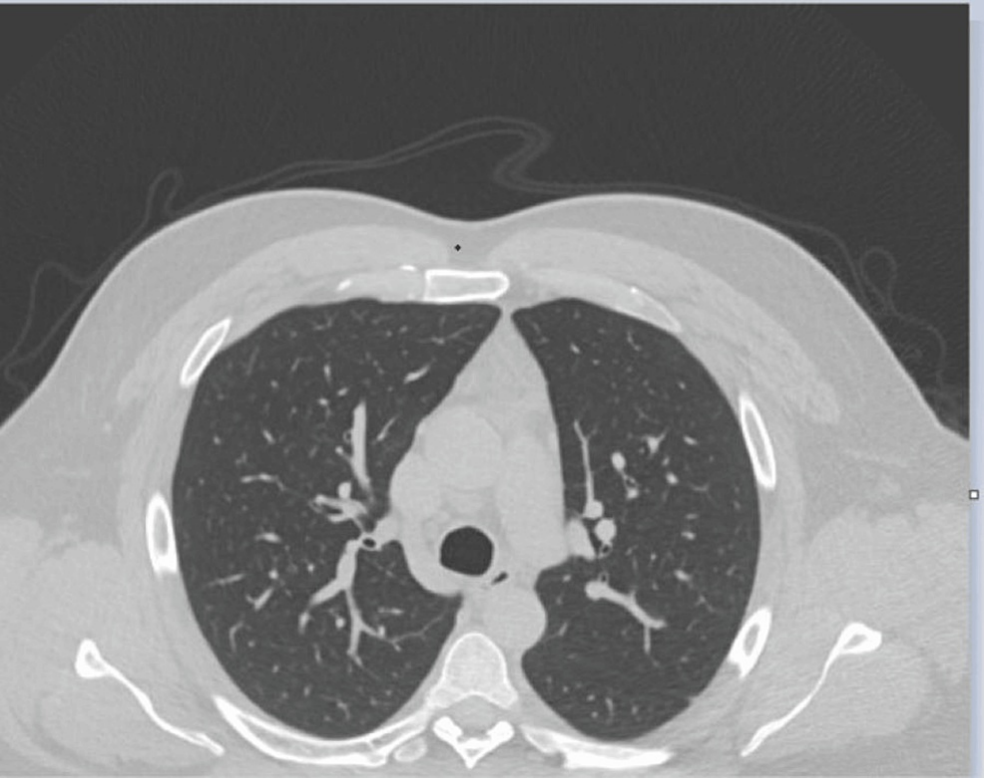
Lung CT image of resolved ground-glass opacities in a case of acute eosinophilic pneumonia (AEP) following a 12-week treatment

Based on the findings, the differential diagnosis included multifocal pneumonia of infectious etiology, acute respiratory failure with hypoxia, and suspected COVID-19 virus infection. Consultations with pulmonology and infectious disease were initiated. From an infectious disease perspective, there was little to no improvement in the patient’s status following changes with antibiotics. Further workup and questioning from pulmonology revealed that the patient had worked in his basement with significant mold exposure three weeks ago and denied the use of recreational drugs and recent travel history. The patient further reported multiple history of incarceration over the years. Per report, it has been more than a couple of years since the last stent and he had never been tested or treated for tuberculosis. Due to the presence of peripheral eosinophil (800 cells/ml) and ground-glass opacities with dense consolidations on bilateral lungs, a bronchoscopy was scheduled. No significant findings on bronchoscopy. Alveolar hemorrhage was absent. Bronchoalveolar lavage (BAL) fluid showed elevated eosinophil at 24%. All cultures were negative. The alpha-fetoprotein (AFP), carcinoembryonic antigen (CEA), and prostate-specific antigen (PSA) levels were within normal limits. Vasculitis autoimmune workup was negative. Noninvasive infectious disease workup was negative. Legionella antigen was absent. Quantitative and qualitative Fungitell was negative. Tuberculosis workup was also negative. The patient was started on steroids with dramatic response and complete resolution of symptoms. Based on this, the patient was diagnosed with acute eosinophilic pneumonia (AEP). A 40 mg Prednisone tablet was started and tapered over a 12-week period. On follow-up appointment early in the fall, ground-glass regions previously seen on CT had completely resolved (Figure 2) and pulmonary function test (PFT) was also within normal limits, although no baseline PFTs were obtained at the initial presentation.

Case 2

In late summer of the same year, there was another case of eosinophilic pneumonia. A 30-year-old male initially presented to the ER with complaints of a worsening non-productive cough that started one week ago. Associated symptoms include rhinorrhea and shortness of breath. His temperature was 98.5F, pulse rate was 90 bpm, respiratory rate (RR) was 20 breaths/min, blood pressure (BP) was 119/77 mmHg, and oxygen saturation (SpO_2_) on room air was 95%. Scattered bilateral wheezing was present in the lungs on auscultation. The patient was diagnosed with acute bronchitis and sent home with albuterol. No further workup or imaging was done at the time.

However, prior to the initial ER visit, the patient had visited his primary care physician (PCP) twice within a three-week period with complaints of shortness of breath and cough that started when he was working around some drywall dust. With each primary care physician (PCP) visit, his vitals were stable and SpO_2_ on room air was 100%. Bilateral lungs were clear on auscultation with good air movement. Heart sounds were within normal limits, with regular rhythm, and rate without murmurs. Chest X-ray showed no acute pulmonary process. COVID-19 test was negative. Based on the physical exam findings, the patient was diagnosed with acute bronchitis due to drywall dust exposure by his PCP. He was prescribed albuterol and a five-day prednisone taper on each occasion.

Seven days after the initial ER visit, the patient returned to the ER with complaints of cough, shortness of breath, with saturations of 90%, tachypnea, and tachycardia. History included recent use of prednisone, use of albuterol without relief, and persistent cough for the last three to four weeks. He denies a history of asthma, pneumonia, and tobacco smoking. The family history was positive for a brother who has asthma. The patient works at a manufacturing plant where they produce polyvinyl chloride (PVC) pipes. Vital signs were as follows: temperature at 99F, pulse rate of 120 bpm, RR of 24 breaths/min, BP at 117/79 mmHg, and SpO_2_ at 94% on 4L/min of oxygen. The pulmonary exam showed that the patient was in respiratory distress with bilateral wheezing, decreased breath sounds, and diffuse inspiratory and expiratory bronchospasm. Complete blood count (CBC) with differentials revealed elevated white blood count at 15.1 K/µl. There was also mildly elevated segmental neutrophil, monocytes, and immature granulocytes. D-dimer was elevated at 0.89 µg/ml, and troponin I was within normal limits. C-reactive protein (CRP) and erythrocyte sedimentation rate (ESR) were elevated at 8.9 mg/L and 30 mm/hr, respectively. Lactic acid and procalcitonin were within normal limits. COVID-19 test and influenza virus test were negative.

**Figure 3:**
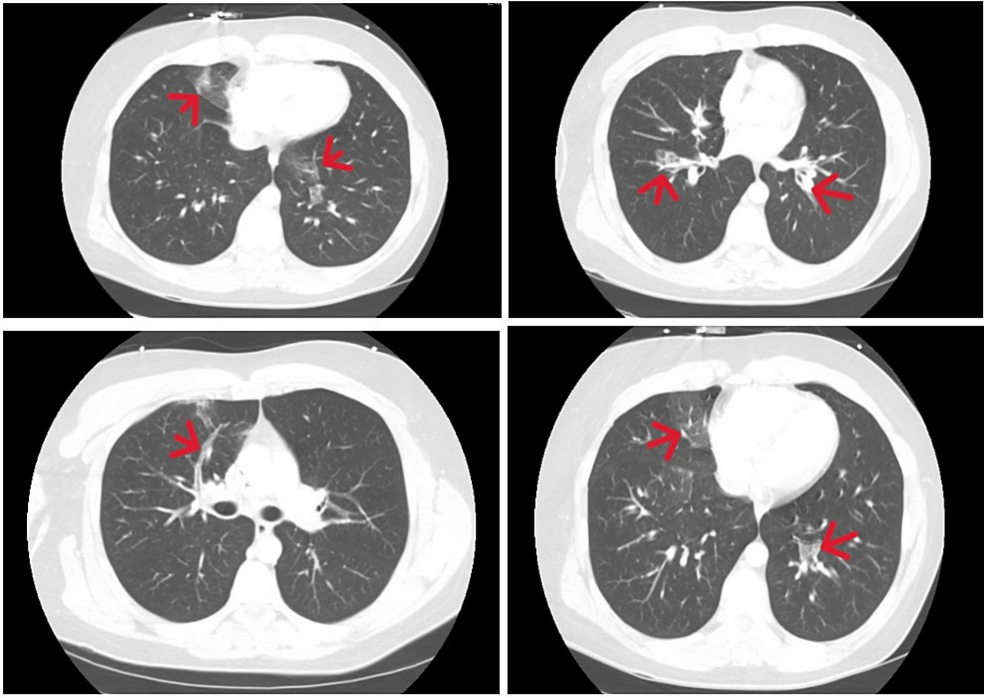
Initial CT scan showing scattered areas of patchy infiltrates (red arrows), in the medial segment of the right middle lobe, the left lower lobe, and the anterior right upper lobe of a patient diagnosed with chronic eosinophilic pneumonia (CEP)

Chest X-ray was unremarkable. The CT chest showed no evidence of pulmonary embolism but showed scattered areas of patchy infiltrates in the medial segment of the right middle lobe, the left lower lobe, and the anterior right upper lobe. There were small areas of ground glass infiltrate on both lungs. The prominent hilar lymph nodes bilaterally were reported as being almost certainly reactive (see Figure 3). Blood cultures showed no growth. Mycoplasma IgM screen was positive.

Following thorough workup findings, the patient was admitted to the floors with broad-spectrum antibiotics and a consultation with the pulmonologist. On day two of the hospital stay, CBC with differentials returned with elevated eosinophil at 1.7K/µl. The pulmonologist considered atypical pneumonia due to positive mycoplasma antibodies, eosinophilic pneumonia, and endemic fungal infection specifically histoplasma because it can lead to peripheral eosinophilia. Other infectious antigen tests were ordered. Quantitative and qualitative Fungitell was negative. Tuberculosis workup was also negative. In addition to antibiotics, the patient was started on Pulmicort nebulizer and IV prednisone and monitored for status change. Bronchoscopy was considered pending patient’s response to the treatment plan. The patient responded swiftly to the treatment, and bronchoscopy was deferred. The patient was discharged with a 15-day course of prednisone taper and a planned eight-week follow-up visit with outpatient pulmonology.

Unfortunately, 16 days after discharge, the patient returned to the ER with worsening cough and wheezing. The patient reported a recent return to work with persistent use of protective gear at work. According to the patient, new symptoms were less likely to be related to PVC exposure from work. The patient reported new smoke exposure from wildfire and poor response to an albuterol inhaler. The physical exam showed oxygen saturation at 95% on room air. There was the presence of wheezing and rhonchi on bilateral lungs. Hematologic findings showed a WBC of 21.8 K/µl, eosinophil of 0.6 K/µl, and lactic acid of 2.6 mmol/L. Procalcitonin, D-dimer, and troponin I were all within normal limits (see Table 2). EKG was reviewed and interpreted as normal sinus rhythm with no obvious ST elevation/depression.

**Table 2:** Hematologic finding seen in a patient with chronic eosinophilic pneumonia (CEP) over the course of first and second hospitalizations ER: emergency room, Seg: segmented, ESR: erythrocyte sedimentation rate, FEU: fibrinogen equivalent unit.

Hematologic findings	Hospitalization day 1	Hospitalization day 2	16 days after discharge with ER return that led to a second hospitalization	Reference range
WBC	15.1 K/µl	18.7 K/µl	21.8 K/µl	4-11 K/µl
Seg neutrophil absolute	11.2 K/µl	12.7 K/µl		1.8-8.0 K/µl
Monocyte absolute	1.2 K/µl	1.5 K/µl		0.0-1.0 K/µl
Eosinophil	0.6 K/µl	1.7 K/µl	0.6 K/µl	0.0-0.7 K/µl
C-reactive protein	8.9 mg/L			0.0-8.0 mg/L
ESR	30 mm/hr		8 mm/hr	0-14 mm/hr
Procalcitonin			0.3 ng/ml	Less than 0.07 ng/ml
Lactic acid	1.1 mmol/L			0.5-2.2 mmol/L
Troponin	0.001 ng/ml		0.009 ng/ml	0.000-0.033 ng/ml
D-dimer	0.89 µg/ml FEU		0.48 µg/ml FEU	Less than 0.49 µg/ml FEU
Bronchoalveolar lavage eosinophil			30%	

CT scan showed moderate multifocal ground-glass air space and interstitial pneumonitis consistent with the inflammatory process or possible aspiration. With the pulmonary team on board, a bronchoscopy was performed. The visualized portion of the trachea showed normal caliber. Bronchoalveolar lavage (BAL) fluid cell count was significant for elevated eosinophil at 30%. Last hospitalization 16 days ago was consistent with an elevated eosinophil at 1.7 K/µl. Both of these findings strongly suggested a diagnosis of chronic eosinophilic pneumonia (CEP). The etiology of CEP such as Loeffler's syndrome was ruled out due to the absence of eating game meat and travel to endemic areas. The patient was discharged on a six- to 12-week high-dose prednisone taper with outpatient pulmonology clinic visits. The patient was also put on trimethoprim/sulfamethoxazole prophylaxis.

With continued outpatient follow-up with pulmonology, the cause of CEP could not be determined. All infectious workups including BAL fluid culture returned negative. Autoimmune workup and antineutrophilic cytoplasmic antibodies (ANCA) were negative. There was an absence of new drugs that could have caused eosinophilic pneumonia. No concern for any parasitic exposure. No vaping history was noted to suggest vaping-induced eosinophilic pneumonia.

**Figure 4:**
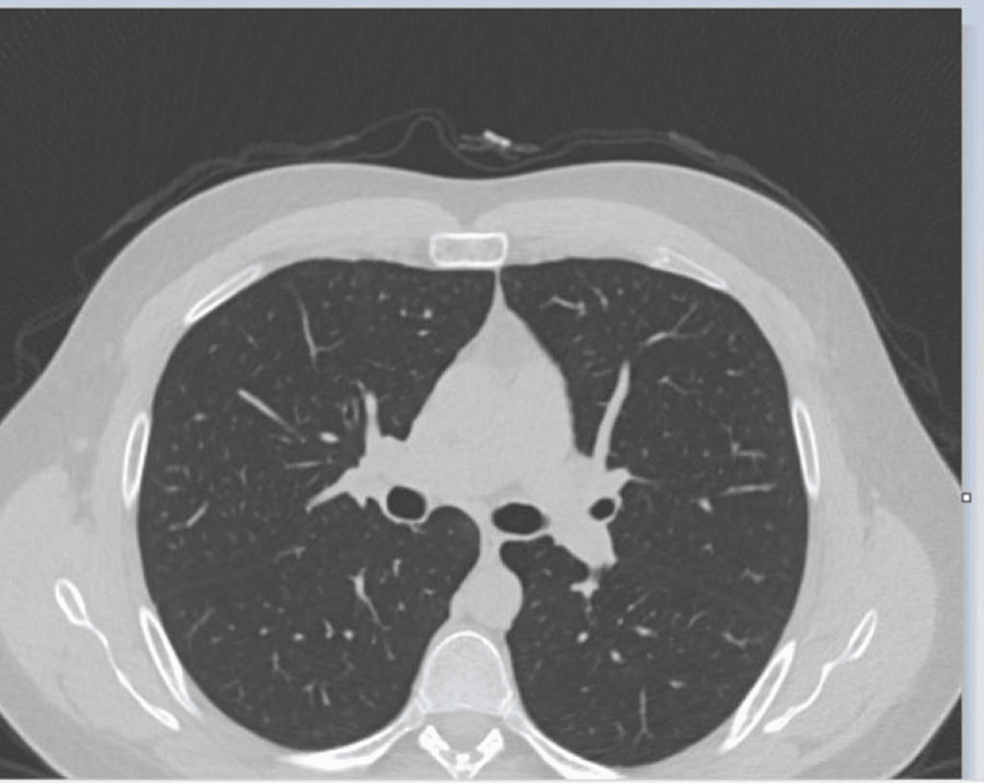
Lung CT image of resolved scattered patchy infiltrates in a patient diagnosed with chronic eosinophilic pneumonia (CEP) after three months of follow-up and treatment

With outpatient follow-ups, the patient complained of intermittent mild symptoms such as chest tightness, wheezing, and shortness of breath. This led to dose changes to systemic corticosteroid taper with the addition of corticosteroid inhalers to the treatment plan. Overall symptoms reported during the pulmonology clinic visit suggested possible asthma of eosinophilic phenotype. The patient maintained long-term care with the pulmonology clinic with the goal to stay on the lowest possible dose of systemic corticosteroid to lower relapse frequency. Three months into his visits with the pulmonologist, a CT scan of the chest showed complete resolution of the previously seen ground-glass opacities on bilateral lungs (Figure 4). Pulmonary function test (PFT) revealed stable lung function and normal spirometry with adequate diffusion capacity.

## Discussion

The incidence of acute and chronic eosinophilic pneumonia is very rare. An epidemiology study estimated 9.1 cases/100,000 person-years with acute eosinophilic pneumonia (AEP) [1,3]. In contrast, the chronic eosinophilic pneumonia (CEP) annual incidence reported by a small retrospective study was estimated to be 0.23 cases/100,000 population [3]. Most patients with AEP are men and tend to be younger between the ages of 20 and 40 years. However, CEP can develop at any age with most patients between the ages of 30 and 50. Women are more likely to develop CEP versus AEP [3,4]. The mean age of CEP diagnosis in women is 45 years old [5].

Several studies have shown the association between smoking habits and the onset of AEP. These studies showed that 50%-80% of patients develop AEP within two months of starting smoking. In addition, changes in smoking habits such as an increase in the quantity of cigarettes smoked or restarting smoking habits resulted in the onset of AEP [3]. In case 1, the patient had quit smoking two years before the onset of AEP; however, he was exposed to mold three weeks before symptoms onset. Hence, other environmental factors in the home, smoke from fireworks, firework dust, indoor renovation, woodpile moving, plant repotting, etc. have been noted to trigger AEP [1,3]. On the contrary, most patients with CEP (>60%) have never smoked [3]. However, a close association between CEP and allergic disease has been noted with more than half of the patients with CEP reporting some type of allergic disease such as allergic rhinitis, atopic dermatitis, and asthma [3,4]. Interestingly, the allergic disease can develop before or after the onset of CEP [3]. In case 2, after the initial diagnosis of CEP, the patient presented to the clinic with multiple symptoms suggestive of asthma, an eosinophilic phenotype, which begins after the onset of CEP in a patient with no previous history of asthma, except for asthma history in a first-degree relation.

Some studies have reported seasonal variations in the occurrence of AEP, with higher case frequency seen in the summer months [1]. No such correlation has been made with CEP. This may be due to its insidious features such as spanning for a longer period, not being able to pinpoint when symptoms first appeared, and late diagnosis of the disease. The seasonal variation could be explained by an increase in unidentified antigens in the air during the warmer months, which are more likely to lead to eosinophilia recruitment, degradation, edema, and subsequently lung damage [1].

Unlike AEP, there are no established diagnosing criteria for CEP [4]. However, certain characteristic features are suggestive of CEP such as ongoing respiratory symptoms for more than two weeks, the presence of bilateral ground-glass opacity distributed peripherally in the middle and lower lungs, BAL >25% eosinophils, and pulmonary tissue biopsy that demonstrates interstitial and alveolar eosinophils (see Table 3) [4,5].

**Table 3:** Diagnosing criteria for acute eosinophilic pneumonia (AEP) and characteristic findings suggestive of chronic eosinophilic pneumonia (CEP) BAL: bronchoalveolar lavage.

Philit diagnosing criteria for acute eosinophilic pneumonia (AEP)	Characteristic findings highly suggestive of chronic eosinophilic pneumonia (CEP)
Acute respiratory illness of less than or equal to one month	1. Greater than two weeks of respiratory symptoms
Pulmonary infiltrate on chest radiography or CT	2. The presence of bilateral ground-glass opacity distributed peripherally in the middle and lower lungs
Pulmonary eosinophils in BAL fluid (or increased percentages of lymphocytes and neutrophils) or eosinophilic pneumonia on lung biopsy	3. Bronchoalveolar lavage >25% eosinophils and pulmonary tissue biopsy that demonstrates interstitial and alveolar eosinophils
Exclusion of other causes of eosinophilic pneumonia	4. Exclusion of other causes of eosinophilic pneumonia

The patient in case 2 demonstrated all these findings, suggesting CEP as the most likely diagnosis. However, other differential diagnoses for CEP can include eosinophilic granulomatosis and angiitis, helminth infection, drug reaction, and allergic bronchopulmonary aspergillosis (ABPA). All these differential diagnoses were ruled out due to the absence of new drug use, absence of parasitic exposure, negative infections workup, and negative ANCA antibody workup. In addition, eosinophilic granulomatosis is usually considered in the presence of extrathoracic symptoms such as arthritis and rash which the patient in case 2 did not present [4].

AEP diagnosing criteria have been constantly modified, and the most recently modified criterion for diagnosing AEP is known as the Philit criteria [1]. According to these criteria, definite AEP is 1) acute respiratory illness of less than or equal to one month, 2) pulmonary infiltrate on chest radiography or CT, 3) pulmonary eosinophils in BAL fluid (or increased percentages of lymphocytes and neutrophils) or eosinophilic pneumonia on lung biopsy, and 4) absence of other specific pulmonary eosinophilic diseases including eosinophilic granulomatosis with polyangiitis, hypereosinophilic syndrome, and allergic bronchopulmonary aspergillosis [1]. The patient in case 1 satisfied the Philit criteria with an extensive workup to adequately rule out other pulmonary eosinophilic diseases.

Both CEP and AEP present differently clinically. Common symptoms associated with CEP include cough, progressive dyspnea, fever wheezing, and night sweats [4]. Cough and dyspnea tend to be seen in most cases [4]. However, systemic symptoms except for fever, fatigue, and weight loss are usually rare in CEP [4]. Limited extrathoracic symptoms such as pericardial effusion, nonspecific skin manifestations, and altered liver function tests have been reported in some cases of CEP [6]. These were not observed in our case of CEP. In contrast, patients with AEP usually present with a swift onset of cough, tachypnea, and dyspnea [7]. Subacute duration of symptoms can also be seen with AEP, but they are usually less common [7]. In cases of AEP, patients can rapidly progress from mild dyspnea to life-threatening respiratory failure within a short time frame [7]. Severe hypoxemia and respiratory failure can be seen with AEP and most patients present with SpO_2_ <90% [3]. Similarly, acute respiratory distress syndrome (ARDS) can present with severe hypoxemia as seen with AEP; however, in the case of AEP, there is the absence of organ failure and shock [7].

Bronchoalveolar lavage (BAL) fluid differential count is an important benchmark in the diagnosis of AEP and CEP. Bronchoalveolar lavage (BAL) is usually done to exclude alveolar hemorrhage and infection in patients with AEP. In both AEP and CEP, BAL fluid differential count is characterized by elevated eosinophilia >25% [1,3,4]. In most cases of AEP, blood eosinophilia is usually within normal limits and tends to rise in the following days [1]. In contrast, elevated blood eosinophilia is mostly observed at initial workup in patients with CEP and is seen in 80%-90% of cases [2-4]. Other expected laboratory findings seen in both AEP and CEP are elevated C-reactive protein and erythrocyte sedimentation rate which are indicative of inflammatory process [3,4].

High-resolution computed tomography (HRCT) is another important tool utilized in the diagnosis of AEP and CEP. The CT findings of patients with AEP differ from those with CEP considerably [7]. In a retrospective study, ground-glass opacity was the most common finding seen on CT in about 97% of cases followed by bilateral pleural effusion (88%) and interlobular septal thickening (68%) in patients with AEP [8]. Air space consolidation is seen in about 40%-60%, and poorly defined centrilobular nodules were noted in 30%-50% of AEP cases [3,8]. Some reports suggest that the presence of bilateral pleural effusion and interlobular septal thickening on CT is highly characteristic of idiopathic acute eosinophilic pneumonia (IAEP) [7]. However, CT findings that are typically seen in CEP include airspace consolidation and ground-glass attenuation [3,7]. The airspace consolidation seen on CT in patients with CEP is predominantly distributed peripherally in the middle or lower lobes as seen in 85% of CEP cases [3,7]. This finding is distinctly different from the CT findings seen with AEP and emphasizes the importance of HRCT in diagnosing the types of eosinophilic pneumonia.

No known factors have been confirmed to predict the relapse rate in eosinophilic pneumonia [3]. Depending on the type of eosinophilic pneumonia, some associations have been made and reported in the literature. Past studies suggested that there were no relapses seen in patients who recovered from AEP; however, present studies have shown that relapse can occur in patients after recovering from AEP [8]. Most cases of relapse reported in patients with AEP were associated with the resumptions of smoking following initial cessation [1,8]. This is not surprising knowing that smoking is highly associated with the onset of AEP. Categorically, relapses are commonly seen in CEP in comparison to AEP. A retrospective study found that patients with CEP and comorbid asthma had lower rates of relapse versus those without asthma (56% vs. 23%) [3]. The explanation for this finding assumed that the higher use of inhaled corticosteroid (ICS) in controlling asthma simultaneously manages symptoms of CEP [3]. Contrary, some case reports have shown that beclomethasone monotherapy has failed to control CEP if used as initial treatment [3].

Corticosteroid remains the mainstay treatment for the management of eosinophilic pneumonia. Patients with AEP respond rapidly to corticosteroids. Conversely, recovery from AEP without steroids has been reported [7,8]. The optimal dose and duration of corticosteroid use in treating AEP have not reached a clear consensus [1]. Depending on presenting symptoms, corticosteroid dose, route, and frequency vary in treating patients with AEP [1,3,7,8]. In the presence of severe respiratory failure, high-dose intravenous administration of corticosteroid is utilized [1,3,7,8]. A study by Rhee and colleagues compared the treatment efficacy of a two-week versus four-week corticosteroid treatment taper in patients with AEP [8]. They found out that the efficacy of a two-week corticosteroid taper was similar to the four-week taper in terms of complete resolution of clinical symptoms and radiographic abnormalities [8]. Treatment of CEP varies significantly from that of AEP. CEP treatment is based on long-term management that focuses on lowering relapse frequency and adequate control of comorbidities [3]. Indefinite use of corticosteroids is employed in the treatment of CEP due to the persistent nature of the disease [3]. Corticosteroids are used for at least three to six to nine months [2-4]. Trimethoprim/sulfamethoxazole for *Pneumocystis jirovecii* prophylaxis is recommended when treating patients with CEP on chronic systemic corticosteroids [4]. Current biologic medications that target interleukin-5 (IL-5) have been approved by FDA to treat eosinophilic disorder [9]. This can be useful in cases of CEP where prolonged doses of corticosteroids are required. Mepolizumab is reportedly being used effectively to manage a patient with CEP [5]. A retrospective study by Brenard and colleagues showed that mepolizumab is effective at reducing the relapse rate of CEP and complete cessation of corticosteroid use [10]. The literature highly supports the use of mepolizumab in the treatment of CEP; however, the duration of treatment is yet to be determined [5,10]. Regardless of the treatment choice, benefits and risks should be considered.

## Conclusions

Eosinophilic pneumonia is a very rare condition, and it seems unusual to have been presented sequentially in two different patients. In both cases, extensive workup had to be done to finalize the diagnosis, following diagnostic criteria for AEP and characteristic features of CEP. High clinical suspicion for AEP versus CEP can be made if clinicians recognize unique differences and similarities in the clinical manifestation of AEP and CEP. Other features that are useful in the successful diagnosis include BAL fluid differential count and recognizable findings on HRCT. Treatment options vary in terms of dose, route, frequency, and duration when treating patients with either AEP or CEP. Relapse should be considered for both AEP and CEP, even though there are higher rates of relapses seen in CEP. Regardless of the type of eosinophilic pneumonia, prognosis is usually excellent if a prompt and accurate diagnosis is made.
